# Biomarkers and Target-Specific Small-Molecule Drugs in Alzheimer’s Diagnostic and Therapeutic Research: From Amyloidosis to Tauopathy

**DOI:** 10.1007/s11064-024-04178-w

**Published:** 2024-06-06

**Authors:** Li Sheng, Rajiv Bhalla

**Affiliations:** Centre for Advanced Imaging, The University of Queensland, St Lucia, Brisbane, QLD 4072 Australia

**Keywords:** Alzheimer’s disease, Diagnostic PET, Drug discovery, Amyloidosis, Tauopathy

## Abstract

Alzheimer’s disease (AD) is the most common type of human dementia and is responsible for over 60% of diagnosed dementia cases worldwide. Abnormal deposition of β-amyloid and the accumulation of neurofibrillary tangles have been recognised as the two pathological hallmarks targeted by AD diagnostic imaging as well as therapeutics. With the progression of pathological studies, the two hallmarks and their related pathways have remained the focus of researchers who seek for AD diagnostic and therapeutic strategies in the past decades. In this work, we reviewed the development of the AD biomarkers and their corresponding target-specific small molecule drugs for both diagnostic and therapeutic applications, underlining their success, failure, and future possibilities.

## Introduction

### Amyloid Cascade Hypothesis

From the age of post-mortem study to the era of non-invasive imaging, AD has been studied throughout the human history of medications. On post-mortem level, there are two pathological hallmarks of AD development: the abnormal deposition of β-amyloid (Aβ) plaques and the intracellular accumulated neurofibrillary tangles (NFTs). The two hallmarks usually appear visibly distinguishable in patients’ brain tissues and have their respective building blocks: Aβ and tau protein. The co-dependence of the two hallmarks is frequently described by the amyloid cascade hypothesis as “the trigger” and “the bullet” [1]. This popular and well-accepted theory of amyloid-triggered tauopathy model has been sufficiently supported by numerous preclinical studies: in vivo studies on genetically-engineered murine models with Aβ-related gene insults reveal that toxic Aβ species stimulates the formation of pathological tau by altering the activities of the protein kinases and phosphatases that regulate tau phosphorylation as well as directly inducing tau misfolding, indicating that the two pathological hallmarks develop codependently [2, 3]. Once pathologically altered, abnormal tau mediates neurotoxicity in many ways, including causing the synaptic dysfunction and neuron death that underlie memory and cognitive impairment in AD patients [1]. Furthermore, the lack of tau expression has been reported to protect against excitotoxicity and prevent memory deficits in mice expressing mutant amyloid precursor protein (APP) identified in familial Alzheimer disease [4]. These various preclinical studies offer significant proof that tau instead of Aβ poses as the direct cause of neurotoxicity in AD cognitive impairment and its alteration is stimulated by Aβ signalling. Their results and conclusions are subsequently summarised in the “amyloid cascade hypothesis” in which Aβ stress induces and mediates neurotoxicity via tau.

So far, the amyloid cascade hypothesis has offers great guidance to the diagnostic and therapeutic developments of AD. In the pathological timeline hypothesised, amyloidosis occurs before the beginning of tauopathy. Clinically in human patients, Aβ deposition may occur even two decades before diagnosed AD. Patients with mild cognitive impairment (MCI) are frequently diagnosed with brain amyloidosis and have a higher chance of developing into AD (10–15% per year). However, Aβ-specific PET imaging studies have also demonstrated that such transformation is not inevitable [5]. This subsequently created a window for early-stage AD diagnosis and AD preventive treatment. As a result, AD diagnostic imaging development has been pursuing two goals: apart from the accuracy to distinguish AD and non-AD dementia, the accuracy to mark the difference between age-related normal MCI and mild AD is also greatly valued. The AD therapeutic development, on the other hand, has gradually expanded the focus on the cognitive improvement of the mild-to-moderate AD patients, to the prevention of the AD-induced MCI patients’ potential progression. In currently on-going clinical evaluations, the new generation of AD diagnostic PET tracers are specifically designed to target tau aggregates to clearly mark the tauopathy-induced neurotoxicity in AD, whilst the efficacy of the recently FDA-approved AD drug leqembi, targeting AD Aβ, was confirmed by clinical trials in MCI and prodromal AD patients.

### AD Diagnostic PET and [^18^F] Fludeoxyglucose

However, histology-based AD identification can hardly benefit the human patients who seek early diagnosis and treatment for their cognitive conditions. As the response, various non-invasive imaging methods such as magnetic resonance imaging (MRI), computed tomography (CT), single photon emission computed tomography (SPECT) and positron emission tomography (PET) have revolutionised the study of AD and liberated the AD pathological research from post-mortem studies into the field of visualised living human organs. Diagnostic PET imaging is widely explored along with people’s search for pathological explanation and therapeutic treatment of the disease due to the same need for efficient biomarkers. As the revelation of AD pathology paves on throughout the past five decades, AD diagnostic PET has evolved past glucose uptake, amyloidosis and tauopathy as the target mechanism for human AD diagnosis. So far, the development of AD diagnostic PET has already offered four generations of PET tracers on its still ongoing road, each of which pushed forward people’s understanding in AD pathology and revealed new obstacles.

**Fig. 1:**
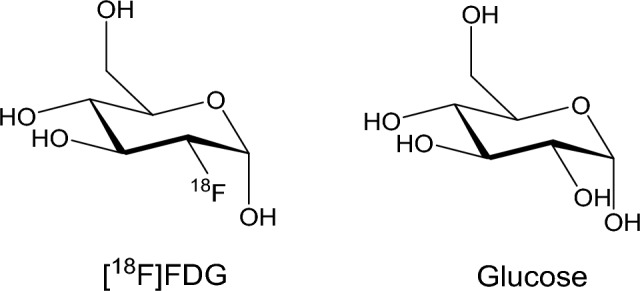
[^18^F]FDG and glucose. [^18^F]FDG was designed and prepared by the fluorination on the 2-OH of glucose, which grants the analogue its entrance to the glucose metabolism in vivo and subsequently marks its path with observable signal for both pathological studies and diagnostic imaging

Brain glucose metabolism is comprised of a series of processes by which glucose is converted into ATP to be used for cellular energy: glycolysis in the cytoplasm, Krebs cycle and oxidative phosphorylation in the mitochondria [6]. The first imaging strategy for AD diagnostic PET in human history was developed in the 1970s and marks AD’s impaired brain glucose metabolism. The drop in the glucose metabolism level was reported to be observable from the very early stage of AD [7, 8], and in the follow-up studies, such change significantly intensified as the patients proceed to a late stage of AD [9, 10], confirming the significance of glucose metabolism level in Alzheimer’s pathological progression. Imaging the glucose metabolism level change was made possible and subsequently became the earliest successful early-stage AD diagnosis method after the introduction of the fluorinated glucose analogue probe [^18^F] fludeoxyglucose (FDG) [11]: a mimic molecule that was originally designed as a glucose probe with its full synthesis routine published in 1969 [12] (Fig. 1). To this day [^18^F] FDG remains as one of the most widely applied PET imaging tracers for AD diagnosis [13].

However, the non-specific nature of the glucose metabolism poses more challenges to the application of [^18^F] FDG as a diagnostic agent. While [^18^F] FDG excels in early-stage AD detection due to its prevalence in diagnosed dementia cases, it also highlights the difficulty in distinguishing non-AD dementia, as altered brain glucose metabolism is shared with other conditions such as Parkinson’s disease and Lewy body dementia [13]. While considering AD’s significantly higher percentage in diagnosed dementia cases, [^18^F] FDG offers excellent accuracy in early-stage AD detection, however, its application significantly adds up to the difficulty of the non-AD dementia identification.

## Aβ as an AD Biomarker

### Aβ-Targeting PET Tracers for AD Diagnosis

**Fig. 2:**
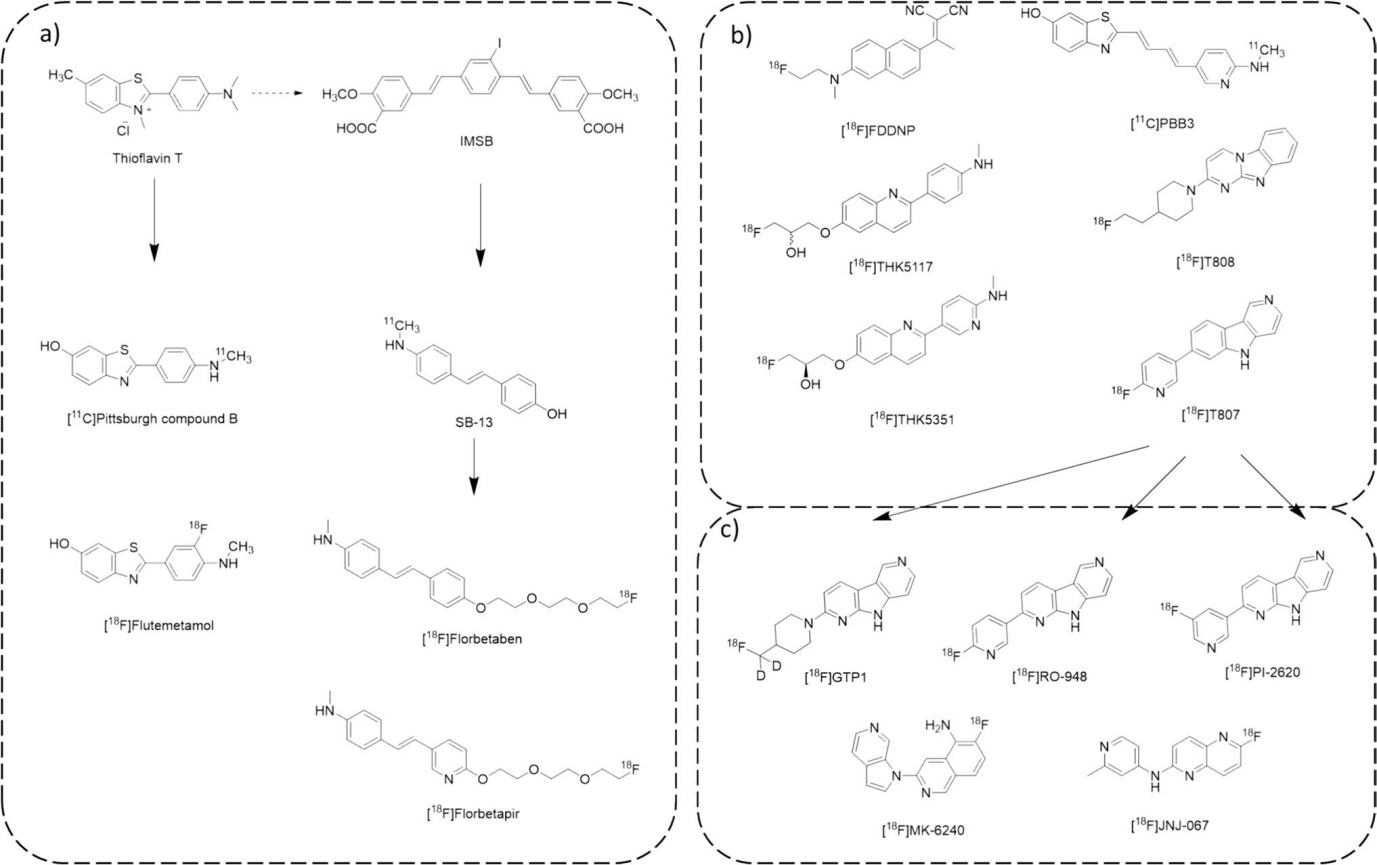
Development of the hallmark-targeting PET tracers for AD diagnosis **a** Aβ tracers **b** first-generation tau tracers **c** second-generation tau tracers

Of the two AD pathological hallmarks, the amyloid plaques’ building block Aβ was the first to be successfully targeted as an AD biomarker in PET tracer development. The development of the current Aβ-PET imaging agents was largely inspired by the post-mortem amyloidosis fluorescent dye thioflavin-T (ThT). In 1960s, ThT was first identified as a useful tool for monitoring AD-related amyloid deposition [14–16] and later became the golden standard for amyloid fibrils staining [17, 18]. The small molecule’s unique ability to recognise the diverse types of amyloid fibrils drew the attention of researchers who were interested in non-invasive imaging agents that monitored the activities of Aβ in vivo. Developed by deriving from the molecular core of ThT, selected from a library of 45 compounds and a short screening of 10^11^C-labelled candidates [19], the ^11^C-labelled benzothiazole derivative [^11^C] Pittsburgh compound B (PiB) (Fig. 2a) quickly became the golden rule tracer for amyloid in vivo identification. The significance of the compound even flourished the tau tracer development that came afterward by offering a foundation for further screening (Fig. 2b, PBB3). The design of [^11^C] PiB molecular structure has replaced ThT’s lipophilic C6-CH_3_ group with a hydrophilic C6-OH. The removal of the positive charge on the benzothiazole nitrogen was a necessary attempt to help the molecule cross the blood–brain barrier. The original C4-dimethylamino group was chosen as the site to dock the radioisotope ^11^C by conveniently methylate the aniline in the late stage of its full synthesis. The first clinical trial of [^11^C] PiB was published in 2004 [20], in which the compound was tested on 16 AD patients and 9 healthy controls. The trial revealed [^11^C] PiB’s significant retention in the areas of association cortex known to contain large amount of amyloid deposits in AD. In contrast, when applied to PD patients, while [^18^F] FDG-PET showed visible retention on both PD patient groups with and without dementia, [^11^C] PiB-PET images displayed no distinguishable difference compared with the control group [21]. So far, [^11^C] PiB has been widely investigated both pre-clinically and clinically, and demonstrated as an excellent tracer for monitoring increased accumulation of amyloid in the brain, however its increasingly wider utility was limited by its poor preservability due to the short half-life of ^11^C (t_1/2_ = 20.3 min). The growing need for fluorinated analogues (^18^Ft_1/2_ = 109.8 min) of [^11^C] PiB led to the development of [^18^F] flutemetamol (also known as GE-067 or [^18^F] AH110690) (Fig. 2a). [^18^F] flutemetamol displays significant molecular resemblance to [^11^C] PiB and behaves similarly to [^11^C] PiB in parallel studies [22]. Published results of [^18^F] flutemetamol’s phase 1 [23] and phase 2 [24] clinical trial both validated its clinical potential as a [^11^C] PiB substitute. In phase 3, [^18^F] flutemetamol-PET was performed a mean of 3.5 months (range, 0–13 months) before the death and autopsy of 68 patients. Sensitivity without computed tomography was 81–93% (median, 88%) and median specificity 88% [25]. In 2013, [^18^F] flutemetamol gained FDA approval under the name Vizamyl.

In parallel to the development of [^11^C] PiB, in 2001, Kung et al*.* published an iodinated styrylbenzene (IMSB) compound which demonstrates an Aβ aggregates-binding ability comparable to ThT [26]. In 2005, Kung et al. subsequently reported the potential stilbene (SB) based compound SB-13 using stilbene as a simplified skeleton substitute for benzothiazole [27] and later in the same year, reported the improved PEG-stilbene series [28]. From their library of SB-based derivatives, lead compound florbetaben (also known as BAY94-9172 and AV-1) and florbetapir (also known as AV-45) became patent-documented along with SB-13 in 2006 [29]. [^18^F] Florbetaben and [^18^F] florbetapir both proved to be ideal [^11^C] PiB substitute in clinical trials [30, 31] and gained FDA approval in 2014 and 2013, under the name Neuraceq and Amyvid respectively.

However, the lack of a control group in the preclinical of PiB was quickly realised and the attempt to fix such problem revealed the false positive in non-dementia patients. [^11^C] PiB-PET imaging study looking at MCI patients instead of PD revealed that subjects with non-dementia MCI have significantly higher [^11^C] PiB uptake in the frontal, parietal, and lateral temporal cortices and the posterior cingulate [32]. As a result, the FDA-approved amyloidosis tracer [^18^F] flutemetamol, [^18^F] florbetaben and [^18^F] florbetapir are catalogued under the instruction to a reduces the likelihood that a patient’s cognitive impairment is due to AD by providing negative scans in order to avoid false AD diagnosis by positive scanning results.

### Aβ-Targeting Small Molecule Drugs

#### APP Secretase Inhibitors

Research in AD therapeutics development have applied different strategies to design small molecule inhibitors that lifts the Aβ stress and prevents the initiation of the amyloid cascade. Currently, the amyloidosis-related AD therapeutics that have been developed and clinically evaluated include two big categories: the γ-secretase inhibitor/modulator and the β-secretase inhibitors. The release of Aβ from the transmembrane APP requires the cleavage of both secretases. First the rate-determining step requires β-secretase to cut APP to generate the membrane-bound C-terminal fragment C99, which is then cleaved by γ-secretase to release Aβ [41]. However, so far, none of these secretase inhibitors/modulators has provided notable cognitive improvement in human objects or received approval for dementia-related treatment. The most concerning event in γ-secretase-targeting drug design is the potential toxicity caused by the unwanted inhibition toward the non-amyloid substrates binding of γ-secretases, particularly the cleavage of Notch, a transmembrane receptor protein family essential to cell differentiation in embryonic development and the dysregulation of which is involved in the malignant transformation in various cancers [42]. In 2008, semagacestat became the first γ-secretase inhibitor to enter phase 3 trial, until the trial was terminated before completion in 2011 due to low efficacy and significant drug-induced side effects, including the elevated skin cancer risk which is believed to be a consequence of inhibited Notch cleavage [43]. Research on the first-in-a-kind Notch-sparing γ-secretase non-inhibitory modulator flurizan was discontinued in 2008 after a multicenter, randomized, double-blind, placebo-controlled phase 3 clinical trial enrolling 1684 patients with mild AD dosing 800 mg twice a day, failed to provide significant results in cognitive improvement [44]. After numerous drug screenings and series of intensive trials that eventually failed, currently, the only active case of γ-secretase modulator is RG6289. In 2023, Roche orally presented the data from the first phase 1 trial of RG6289 [35]. Later in the same year, a PK/PD modelling study in human volunteers for phase 2 dose selection was made available online as a digital poster [45]. A variety of BACE inhibitory small molecule drug candidates stepped forward soon after the failure of the pioneering γ-secretase inhibitory drug candidates. During the past decade, most of the BACE inhibitors also have dropped out of the clinical trials either due to low efficacy or side effects. Currently the only active case is lenalidomide. As an alternative to thalidomide, the BACE inhibitor is hoped to put an end to the severe dose-limiting toxicity of thalidomide and its phase 2 trial is expected to reach primary completion by the end of 2023. However, this trial enrols amnestic mild cognitive impairment patients instead of AD. Nevertheless, various major BACE inhibitors including lanabecestat, umibecestat, verubecestat etc*.*, have reported drug-induced reginal brain volume reduction and this phenomenon has been reviewed as class-specific [46]. The intensive research and the failure of the secretase modulators/inhibitors points out the potential flaw and limit in the amyloid cascade hypothesis. The concept of drug design based on the amyloid-regulated tauopathy, and subsequent tau-mediated neurotoxicity described in the hypothesis, is being challenged and questioned.

#### Other Mechanisms

Currently, several subclasses of non-secretase-targeting small drug candidates are still being studied and evaluated. This includes (1) the ApoE enhancers bexarotene and CS6253, which aim to elevate brain ApoE level to aid in brain Aβ clearance (2) the synaptotoxicity reducer such as simufilam, BMS-984923, and CT1812 attempting to reduce Aβ signalling via different mechanisms (3) the so called “metal protein attenuating compound” PBT series clioquinol (PBT-1) and PBT-2, which prevent the Aβ-metal interaction to reduce Aβ level and restore zinc/copper homeostasis in the brain. Additionally, a variety of AD drugs with independent mechanisms are also listed in Table 1. The upcoming outcomes of their clinical trials may shed light on the future of the anti-Aβ therapeutics and amyloid cascade hypothesis.

**Table 1:** Evaluation of Aβ-related AD small-molecule drugs

Name	Molecular structure	Mechanism	Status
γ-secretase inhibitors/modulators			
Avagacestat		γ-secretase inhibition	Phase 2 trial aborted in 2012 due to strong gastrointestinal and dermatological side effects
Semagacestat		γ-secretase inhibition	Phase 3 trial displayed cognitive worsening with increased risk in skin cancer and infection. Halted in 2011
CHF 5074		Multi-target γ-secretase modulator	Withdraw phase 2 trial in 2012. Case remains inactive^a^
EVP-0962		γ-secretase modulator	Completed phase 2 trial in 2013 then discontinued^a^
Flurizan		γ-secretase modulator	Failed phase 3 trial with negative outcomes in 2008 due to insufficient brain bioavailability
D-Pinitol		γ-secretase modulator	Completed phase 2 trial in 2014. Case remains inactive^a^
PF-06648671		γ-secretase modulator	Discontinued in 2018 after phase 1 trial completed in 2016. Results published in 2020 [33]^a^
RG6289 [34]		γ-secretase modulator	Phase 1 trial completed by 2023 [35]. Results not published
β-secretase (BACE) inhibitors			
Atabecestat		BACE inhibition	Discontinued in 2018 after phase 2/3 trial due to negative results and liver toxicity
BI 1181181		BACE inhibition	Discontinued in 2015 during phase 1 trial^a^
Elenbecestat		BACE inhibition	Discontinued in 2019 during ongoing phase 2 trials due to unfavourable results
Lanabecestat		BACE inhibition	Discontinued in 2018 during phase 3 trial due to negative outcomes
LY2886721		BACE inhibition	Phase 2 trial halted due to toxicity concerns in 2013
LY3202626		BACE inhibition	Discontinued in 2018 after aborting phase 2 trial. Published results concluded no significance cognitive improvement [36]
PF-06751979		BACE inhibition	Discontinued in 2018 after phase 1 trial completed in 2016. Results published in 2019 [37]^a^
RG7129		BACE inhibition	Discontinued in 2013 after phase 1 trial^a^
Thalidomide		Multi-target, BACE inhibitor	Severe side effects and one death reported in first trial. Succeeded by lenalidomide
Lenalidomide		Alternative to thalidomide	Phase 2 trial complete in 2023 concluded its safety and a second trial focusing on biomarker endpoints is scheduled [38]
Umibecestat		BACE inhibition	Discontinued in 2019 during phase 2/3 trial announcing worsened cognition
Verubecestat		BACE inhibition	Discontinued in 2018 during phase 3 trial. Developer announced cognitive worsening
ApoE enhancers			
Bexarotene		Enhance apolipoprotein E (ApoE) particle formation which regulates Aβ metabolism and clearance	Finished phase 2 trial in 2014 identifying reginal amyloid reduction only in ApoE4 noncarrier subgroup and significant risk in blood lipid level elevation
CS6253		Increasing activity of adenosine triphosphate-binding cassette transporter A1 (ABCA1) whose function loss attributes to ApoE reduction in AD	Phase 1 trial still ongoing in 2024
Aβ toxicity reducer			
CT1812		Reduce oligomeric Aβ receptor binding by regulating sigma2 receptor	In phase 2 trial
Simufilam		Target filamin, a stabilizer of Aβ42 and tauopathy upstream regulator α7nAChR	In phase 3 trial
Alzhemed (drug) Vivimind (nutrient)		Glycosaminoglycan mimic molecule that prevents Aβ aggregation’s β-sheet formation by antagonizing Aβ-glycosaminoglycan interactions	Failed to provide cognitive improvement in phase 3 trial in 2007. Currently a commercially available nutrient in Canada
ALZ-801		Prodrug of Alzhemed Aβ42 aggregation inhibitor	A long-term extension of phase 3 study was announced in 2024
BMS-984923		Reduce synaptotoxicity of Aβ oligomers by inhibiting co-receptor mGluR5	In phase 1b trial
Metal protein-attenuating compound			
Clioquinol		Attenuating Aβ peptide-metal interaction	Succeeded by PBT-2
PBT-2			In phase 2 trial, no significant change of Aβ level was observed by PiB-PET
Other mechanisms			
Acitretin		Increase non-amyloidogenic α-secretase of APP	Finished phase 2 trial in 2013. Results published in 2014 [39]^a^
Buntanetap		APP mRNA translation blockage	Phase 2/3 trial recently announced its completion in March 2024. Results not released
ELND005		Neutralizing low-N Aβ oligomers	Completed phase 2 trial in 2015 then repurposed for Down syndrome and bipolar disorder
GV-971		Aβ de-aggregation	Approved in China. US phase 3 trial suspended in 2022 due to financial and pandemic problems
PRI-002[40]	Unpublished	Aβ42 monomer stabilization	Completed phase 1 trial in 2022. Phase 2 trial scheduled for 2024
Varoglutamstat		Reducing pathogenic pyroglutamate Aβ generation by glutaminyl cyclase inhibition	In 2024, developer announced their phase 2b trial failed to meet endpoints

^a^There are no official announcements made behind the inactive or discontinued cases

## From Amyloidosis to Tauopathy

NFTs are insoluble polymerised fibres of altered tau proteins that are found heavily aggregated intracellularly in AD histology. Propagating and maturing withing neurons, NFTs deposition are believed to be directly related to the neurodegeneration progress and the cognitive decline that follows. Its building block tau is a small amyloidogenic protein with six isoforms generated by mRNA alternative splicing. Although leakage of tau from brain interstitial fluids into plasma compartments in AD patients has been observed and reported [47], tau is generally considered as a brain-specific neuronal protein exclusively essential to the brain functions. As a neuronal microtubule-associated protein (MAP), in normal brains tau serves to stimulate tubulins’ assembly into microtubules intracellularly [48]. All tau isoforms are highly soluble and have two domains: the projection domain and the microtubule binding domain. The projection domain which composes two thirds of the protein molecule has two regions: the proline-rich region, and the amino-terminal region that interacts with other molecules with its high portion of acidic residues. The microtubule binding domain can be divided into the basic tubulin binding region and the acidic carboxyterminal region. Defined by the length of the amino-terminal and microtubule binding region, these six isoforms are referred to as 0N3R, 0N4R, 1N3R, 1N4R, 2N3R and 2N4R tau. 0N, 1N and 2N are defined by the number of amino-terminal domain inserts encoded by exon 2 and 3. 3R and 4R are defined by the number of the highly similar repeats of the microtubule binding domain region encoded by exon 9–12, whereas the second repeat (R2, exon 10) is missing in 3R taus [49]. Since they differ in size, these isoforms are often referred to as tau-352, tau-381, tau-383, tau-410, tau-412 and tau-441 respectively by the number of residues. The repeats in the microtubule binding domain regions are believed to be an important factor in dementia-related tau self-aggregation. While evidences suggest an asymmetric barrier between the seeding of 3R and 4R tau [50, 51], however, unlike progressive supranuclear palsy (PSP) and Pick’s disease (PiD) whose NFTs assembly visibly favours 4R and 3R tau respectively [52], AD tauopathy does not display an established preference. In fact, 3R/4R tau aggregates seeding has been observed in the very early stage of AD development [53]. So far, the role of different tau isoforms in AD pathology remains unclear. While the amount of 4R and 3R taus are dynamically equivalent in normal human brains, the most abundant type is the human adult CSF tau-441, which subsequently becomes a significant probe in many AD diagnostic and therapeutic evaluations.

**Fig. 3:**
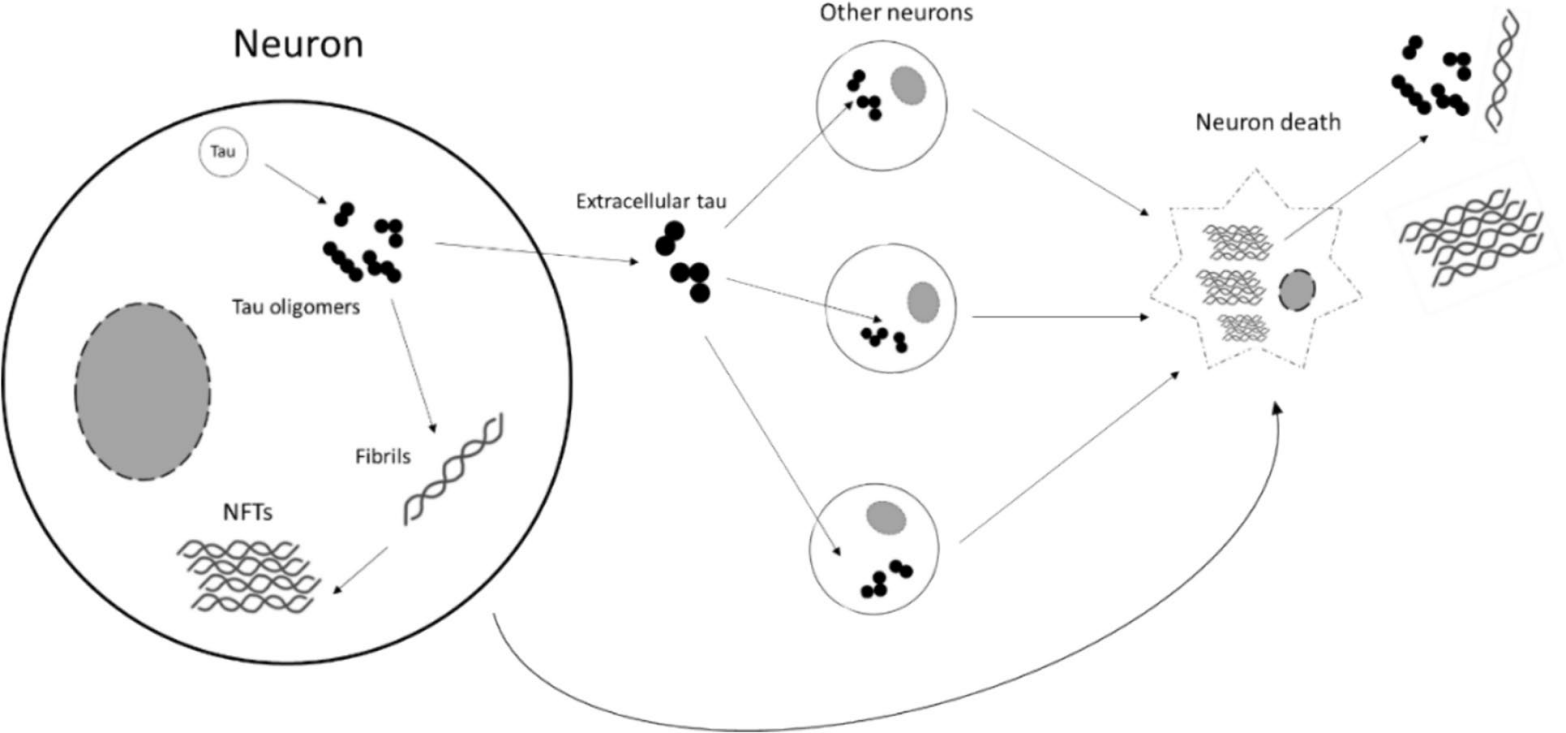
Seeding, growth, and accumulation of NFTs. Hyperphosphorylated taus with elevated self-affinity aggregate to soluble oligomers which may remain in or travel to other neurons to seed fibrils. Polymerised fibrils later build into insoluble intracellular NFTs. These tau aggregates are subsequently released after the neuron death to further spread tauopathy

It has been debated that the neurotoxicity of the NFTs is more of an outcome of the soluble tau oligomer seeding rather than the mature insoluble depositions of the aggregate itself. Before the formation of paired helical filaments (PHFs) and the assembly of NFTs, pretangle tau aggregates, later known as the tau oligomers, were reported present in the earliest stage of AD brains [54]. These oligomers are complexes of more than two, commonly three to ten hyperphosphorylated and normal tau monomers. In the oligomer-nucleated model, the tau oligomers seed and propagate tau aggregates in a prion-like autocatalytic way. Fibrils seeded by oligomers can subsequently offer a template to which more taus are attached until they grow into longer filaments. The cause of tau oligomerisation remains unclear, however, a highly stable, degradation-resistant, insoluble and truncated tau nucleus of 92–95 amino acids ending at glutamate 391 has been identified as PHF cores that sequester phosphorylated and non-phosphorylated taus intact, later growing into PHFs [55]. PHFs can further assemble into NFTs via polymerisation. While PHFs and NFTs are insoluble and immobilised intracellularly, the extracellular tau species plays an active part in spreading tauopathy and neurotoxicity to other brain regions as a result of its ability to exit and re-enter neurons. In 2013, Holmes et al*.* proposed that tau oligomers, rather than monomeric tau, can be internalised by neuronal cells and such uptake is mediated by heparan sulphate proteoglycans that require the binding domain consisting of a stretch of positively charged lysines or arginines on the tau ligand (Fig. 3) [56].

In the cells to which pathological tau species are spread, tau demonstrate its neurotoxicity in multiple ways that disrupt the neuronal cell normal functionalities, including causing the loss of mitochondrial membrane potential [57] and ectopic cell cycle re-entry [58]. Such disruptions are directly associated to the brain synaptic dysfunction: the initial target and key factor of AD that underlies the memory loss [59]. In amyloid cascade hypothesis, neurotoxicity caused by tauopathy is considered as the downstream regulation of amyloid deposition. However, the lack of correspondence between the Aβ deposition map and the brain regions of the highest NFTs level and neuron death [60] points out the limitation of amyloid cascade hypothesis that stress Aβ’s role as an upstream regulator of AD tauopathy. Although the hypothesis remains well-accepted, more and more studies have been demonstrating the activity of tau independently from the brain amyloidosis. With the development and the approval of various non-invasive tau imaging tracers specifically targeting various tau species, in the near future, the monitoring of the human AD tauopathy in vivo may significantly contribute to more revelations in the true nature of the tau-amyloid co-dependency.

## Tau as an AD Biomarker

### Tau-Targeting PET Tracers for AD Diagnosis

As one of the two pathological hallmarks of AD, the development of tau-specific tracers took place during the early years of the Aβ-specific tracer discoveries and thrived considerably after the revelation of Aβ’s accumulation in non-AD dementia and mild cognitive decline patients. One of the earliest tau-targeting tracers, 2-(1-{6-[(2-[^18^F] fluoroethyl)(methyl)amino]-2-naphthylethylidene)malononitrile ([^18^F] FDDNP) was initially designed as a potential Aβ-targeting PET agent, but surprisingly showed combined affinity to both Aβ and tau [61, 62] (Fig. 2b). [^18^F] FDDNP has been used to track tauopathy-related activities in chromic traumatic encephalopathy (CTE) brains [63, 64] and was briefly introduced to AD diagnosis PET with relatively low imaging efficiency [65]. As the co-dependence between AD amyloidosis and tauopathy become clearer, the growing need to understand tauopathy independently from amyloidosis in dementia pathology resulted in the focus on the development of highly tau-specific PET tracers.

#### Tau Tracers: The First Generation

Tau deposits’ different isoform composition, histopathology and ultrastructure in various neurodegenerative conditions has made the development of highly tau-specific agent a very challenging task. As a result, the first-generation tau tracers (Fig. 2b) have all displayed off-target/nonspecific binding to the different degrees. (1) The [^11^C] PiB molecule influenced agent [^11^C] PBB3 developed by the National Institute of Radiological Sciences in Chiba, Japan [66, 67], as a product of PiB-based screening, has unsurprisingly shown to bind dense core amyloid plaques [68]. (2) The ^18^F-labelled 2-arylquinoline derivative series developed by Tohoku University in Sendai, Japan [69] first introduced the lead candidate THK5117. However, the developers soon realised that THK5117 substantial off-target binding to white matter [70] would lead to possible misinterpretation and developed THK5351 to specifically reduce the white matter signal [71]. In their later studies however, the micro-autoradiography demonstrated [^3^H] THK5351 still binds to thread-like structures in white matter [72]. (3) The ^18^F-labeled pyrido-indole compound series developed by Siemens Healthcare provide T807 (also known as AV-1451 or flortaucipir) and T808 as the series’ two outstanding candidates [73–75]. It was subsequently reported that [^18^F] T807 may generate non-specific signal by binding to pigmented and mineralised structures [76]. T808, on the other hand, dropped out due to in vivo defluorination [77].

The shared off-target binding efficacy to monoamine oxidase A (MAO-A) and monoamine oxidase B (MAO-B) of more than one tracer in the first generation came out as a surprise. The oxidase pair are members of the MAO family, a family of enzymes that oxidise monoamines into aldehydes and are usually found bound to the mitochondrial outer membrane [78]. Off-target signal in the MAO-B-rich basal ganglia was reported consistently for both THK5351 [72] and T807 [79]. An inhibitory competition experiment using unlabelled THK5351 and T807 in both hippocampus and putamen homogenates showed that both tau agents displayed off-target binding to MAO-B with affinity comparable to MAO-B inhibitor deprenyl [80, 81]. Tau PET AD imaging using THK5351 can be efficiently correlated by dosing MAO-B inhibitor lazabemide to reduce MAO-B off-target signal [82]. T807 was reported to bind to both MAO-A and MAO-B alike [83], and also unlike THK5351, experiments conducted on rodent models demonstrated that MAO-B inhibitors including lazabemide and moclobemide could not block T807’s standardised uptake value (SUV) while deprenyl can successfully reduce its SUV by binding competition [84]. Additionally, MAO-A selective inhibitor clorgyline was also reported to show positive inhibition of T807 binding [83, 85]. These results may indicate that T807 binds to MAO-B through a mechanism different from that of THK5351. And since the activities of MAOs are mostly irrelevant to AD pathology, the off-target binding to these oxidases becomes an obstacle that needs to be overcome in the development of a new generation of tau-specific imaging tracers.

#### Tau Tracers: The Second Generation

The development of the next generation of AD diagnostic PET tracers is the answer to the unexpected MAOs off-target binding incident. As a result, the second-generation tracers were specifically designed to reduce MAO-A and MAO-B affinity. So far the most promising second-generation tau tracers include [^18^F] MK-6240, [^18^F] RO-948, [^18^F]-GTP1 [86] [^18^F] PI-2620, [^18^F]-JNJ-067 etc. (Fig. 2c). [^18^F] MK-6240 is currently the most studied second-generation tau agent and its high binding affinity for NFTs with significantly weaker MAO-B binding and no binding affinity for MAO-A has already been confirmed [85]. Whilst the preclinical analysis shows promising result for AD tau, recent observation by Aguero indicates that MK-6240 shows strong off-target binding to neuromelanin and melanin-containing cells [87]. [^18^F] RO-948 [88], [^18^F] PI-2620 [89], [^18^F]-JNJ-067 [90], and [^18^F]-GTP1 [91] have so far been characterised in vitro, validated in human patients, reported to successfully avoid off-target binding to the MAOs and currently in clinical evaluations (Table 2).

**Table 2:** Evaluation of the reported tau tracers

Name	Target binding	Off-target/non-specific binding in human brain tissues	Status
[^11^C]PBB3	NFTs, neuropil threads, and plaque neurites	Senile plaques, particularly dense core Aβ plaque	Affected by Aβ binding
[^18^F]THK5117	PHF-tau, intracellular and extracellular tangles, but not pretangles	White matter	Significant nonspecific binding
[^18^F]THK5351	PHF-tau, NFTs	Thread-like structures in the white matter, MAO-B	Off-target signal
[^18^F]T807	PHF-tau	MAO-A, MAO-B	FDA approved
[^18^F]T808	PHF-tau	MAO-B	In vivo defluorination
[^18^F]MK-6240	NFTs	Neuromelanin and melanin-containing cells	Early clinical phase 1
[^18^F]RO-948	NFTs, neuropil threads	Not reported	Early clinical phase 1
[^18^F]-GTP1	NFTs	Not reported	Completed co-analysis in the phase 2 trial of semorinemab [92], then put into phase 1 with [^18^F]MK-6240 and [^18^F]RO-948
[^18^F]PI-2620	NFTs	Not reported	Phase 3 to be completed in 2026
[^18^F]-JNJ-067	Aggregated tau but not 4R tau	Not reported	Completed phase 1 trial in 2021. One of the AD subjects was visually negative [93]

Notably, due to the variety of tau aggregates’ physical forms, the tau tracers developed and evaluated all provide specific binding affinity to different tau species: either NFTs or the PHFs. As tauopathy develops, it has become clear that the neurotoxicity of tau species varies significantly in AD pathology. It would benefit both potential patients and dementia pathologists to be able to visualise major tau species independently. Moreover, if made available, the visualisation of pretangle deposition would significantly benefit the tauopathy research, and early-stage AD identification in the future.

### Tau-Targeting Small Molecule Drugs

#### Tau Aggregation Inhibitors

Tau imaging provides researchers with better means to directly monitor the accumulation of brain taus in vivo and the opportunity to further explore into the possibility of utilising tauopathy as a therapeutic target for AD treatment. The challenges of tau-specificity in tau tracer development also exist in the development of tau-targeting therapeutics. To treat AD-directed tauopathy, many small molecule drugs that aim to prevent the aggregation of tau into complexes and the assembly of tau fibrils have been developed and trialled. These tau aggregation inhibitors (TAIs) can also be divided into two generations. The first generation of TAIs can covalently bind to tau residues via chemical bonds, such as oleocanthal that forms imines with tau’s lysine residues [100] and dimethylfumarate that reacts with cysteine sulfhydryls [101]. However, these covalent inhibitors are troubled by low efficiency in vivo where the chemical environment is more complicated and abundant with protein residues, such as intracellular sulfhydryls in the form of glutathione [102]. The second generation of inhibitors interfere tau aggregation progress through mechanisms that does not involve covalent binding to the residues of tau. For example, aggregation inhibitor baicalein may inhibit heparin-induced tau aggregation by initializing tau oligomer formation. This polyphenol then dissolves these oligomers to prevent their further fibrillization [103]. Tau’s assembly into parallel stacks of β-sheets is necessary for its propagation and neurotoxicity, yet non-covalent inhibitors rosmarinic acid may block the formation of the steric zipper structures essential for the β-sheets folding in order to slow down such progress [104].

Similar to amyloid tracer [^13^C] PiB and tau tracer [^13^C] PBB3, the first identified and the most studied second generation TAIs are derived from a small dye compound methylthioninium chloride (MTC). Also known as methylene blue, the dye was previously applied in the indications of methemoglobinemia, ifosfamide-induced encephalopathy, thyroid surgery etc. [105]. One of MTC’s outstanding advantages is its ability to successfully bypass the blood–brain barrier via oral administration [106]. The proposed mechanism of MTC inhibition is its act of mediating sulflhydryl oxidation of cysteine residues to keep tau monomeric [107]. Like the first-generation tau-targeting tracers, MTC also interacts with both MAOs as a small molecule inhibitor. It is proved to be a fine MAO-A binding inhibitor with IC_50_ = 164 ± 8 nm and a casual MAO-B binder with IC_50_ = 5.5 ± 1.7 μM [108]. MTC is reported to show IC_50_ = 0.15 μM for the dissolution of PHFs isolated from AD brain, while its EC_50_ to cause 50% inhibitions of microtubule assembly and tau expression level are 50 μM and 10 μM respectively [109], leading to the benign conclusion that using MTC-based TAIs as AD therapeutics is unlikely to disrupt normal tau functionality. Nevertheless, in vivo results of MTC activities are rather limited as its staining ability strongly persists. It is hence replaced by Leuco-methylthioninium (LMT), a reduced form of MTC soon afterwards. In 2013, TRx0237 (product name LMTX) developed by TauRx Therapeutics became the first tau aggregation inhibitor to enter phase 3 trial. The stabilised polymorph with LMT as the major active ingredient, can be prepared through bulk manufacture and shows remarkable oxygen stability that synthetic, pure LMT lacks [110]. However, after dosing 891 patients with mild-to-moderate AD with 75 mg or 125 mg of LMTX or placebo twice per day for 15 months, the drug failed to show significant impact on patients’ cognition [111]. In 2020, *Melissa officinalis* extract richly containing the second-generation TAI rosmarinic acid as an active ingredient was trialled for the prevention of AD progression instead of cognitive improvement. The randomized placebo-controlled double-blind 24 week trial on 23 patients diagnosed with mild AD dosed with a daily amount of *Melissa officinalis* extract equivalent to 500 mg rosmarinic acid was met with a similar end of no significant difference in cognitive measures. However, a mild improvement in neuropsychiatric symptoms such as agitation, was observed [94].

The poor clinical performance of small molecule TAIs points back to the acute toxicity of tau pretangles and their remarkable impact on neuron death and cognitive decline than the mature fibrils. Notably, human neurons begin the degeneration process before the formation of NFTs and may also survive the co-existence with NFTs for decades [112]. In fact, the incubation of tau oligomers isolated from human AD brains successfully induces the aggregation of monomeric recombinant tau in vitro [113]. Moreover, intracerebral administration of soluble recombinant tau oligomers prepared in vitro to wild type rodent model leads to dementia-like behavioural deficits [114]. The key role and the notable toxicity of the small soluble tau oligomers with 3–10 repeats may explain the inability of potent TAIs to impact the cognitive decline. In an AD brain where brain taus are highly overexpressed, preventing the coupling of 3–10 tau monomers into oligomers would require excellent drug potency and even higher dose, both of which may also pose as a threat to microtubule assembly. Adapting this rationale, OLX-07010, a small molecule drug candidate developed by Oligometrix, was claimed to be an efficient tau self-association inhibitor which involves in and prevents the initiation of tau aggregation in htau mice [115]. Its first phase 1 clinical trial is completed in July 2023 however, so far, the results have not been made available to the public. The other tau aggregation inhibitor recently in clinical evaluation is ACI-3024. Developed by AC Immune, te small molecule drug is claimed to be an NFT disaggregation agent however with no preclinical data available to the public to demonstrate its mechanism background.

Nowadays, the focus of tau aggregation intervention mainly lies within the field of immunotherapy, with a great number of therapeutic agents currently in clinical trial. To offer an alternative solution to the small molecule inhibitors, researchers have been exploring the possibility of applying TAIs in combined therapies to balance its shortcoming but so far was not met with any breakthrough. In recent years, drug screening assays and SAR evaluations of dual inhibitors that interfere both tau and amyloid aggregation alike have been introduced [116]. In the review article published in 2021, Malafaia et al. summarised the three families of framework-based medicinal chemistry drug screenings that were explored in the search for potent dual-inhibitors. These three base compounds include curcumin, huprine and tacrine [117]. The novel concept of multi-target aggregation inhibitor may serve as the new direction for the hallmark-targeting AD therapeutics.

#### Tau Kinase Inhibitors

To clear the oligomers, it is necessary to explore into the pathological taus’ alteration in AD brains. Although reducing the expression level of tau in transgenic mice model can significantly decrease neuronal cell loss and improve memory loss [118], leading to the conclusion that the assembly of tau oligomers would require concentrated brain tau, normal tau actually does not aggregate in vitro without alterations or the addition of reagents. While purified normal tau usually contains 2 to 3 mol of phosphate per mole, AD taus typically contain 5–9 mol of phosphate per mole of the protein [119]. In vitro studies show that phosphorylation of Thr 231, Ser 235 and Ser 262 residues on tau can lead to inhibition of tau’s microtubule binding by 26%, 9% and 33% respectively [120]. Using chromosome 17 (FTDP-17) mutation murine models, it has been revealed that the mutant tau shows significantly faster phosphorylation on Ser 396, Ser 400, Thr 403 and Ser 404 residues, and observed the mutant type’s quicker self-assemble into filaments compared with the wild type. In addition to their reduced affinity for their original target, these hyperphosphorylated tau displays significantly enhanced affinity for normal taus, leading to the sequestering of taus and disrupt the normal microtubule assembly [121] which further broadens tau’s neurotoxicity impact in AD brains.

**Fig. 4:**
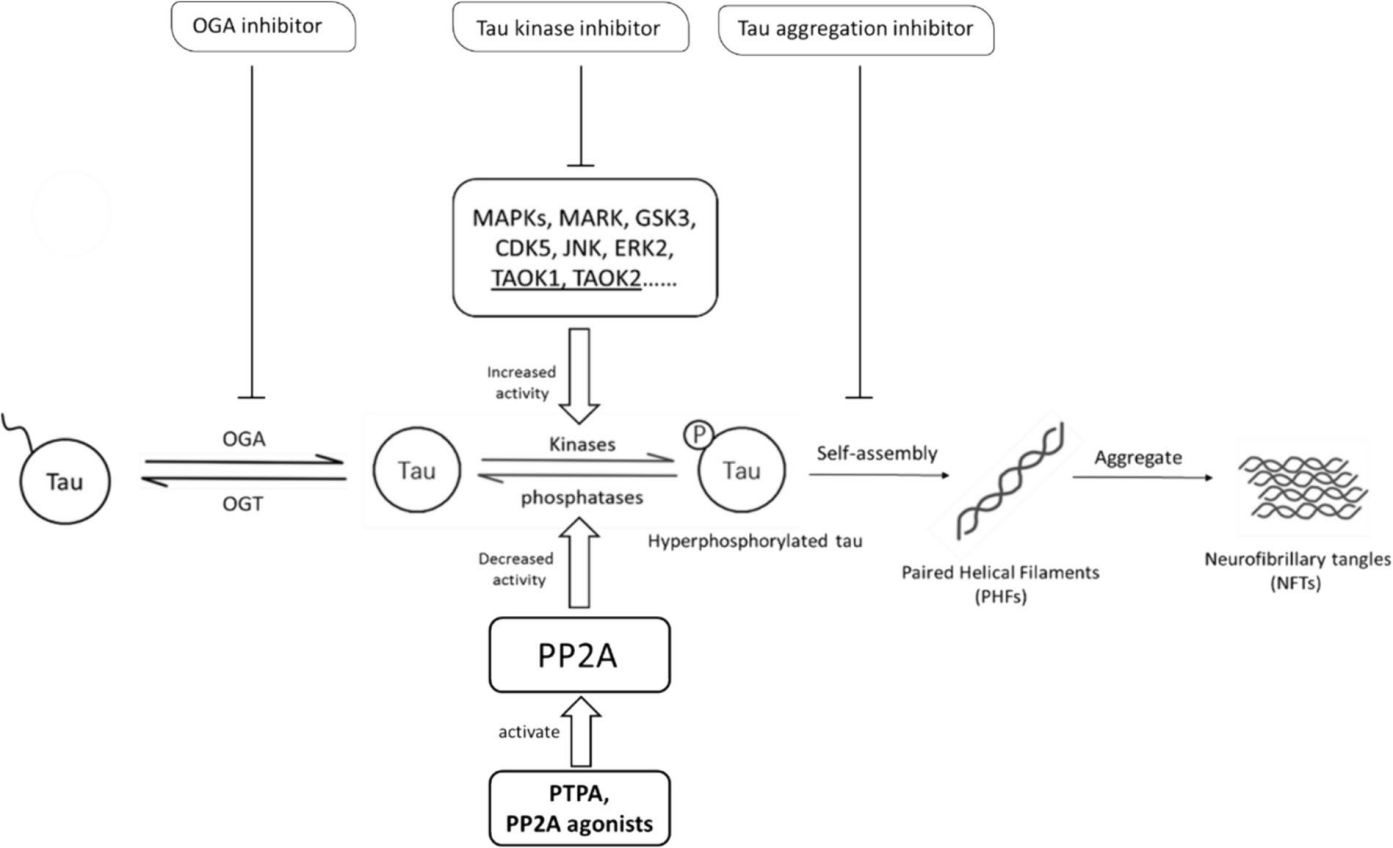
AD-related tauopathy. Based on the hyperphosphorylation, dephosphorylation, propagation and O-GlcNAcylation of tau, various tauopathy-targeting AD therapeutics have been developed

The level of tau phosphorylation is the consequence of the dynamic regulation of tau kinases and tau phosphatases [122]. The tau kinases can be divided in to three classes: proline-directed protein kinases (PDPK), protein kinases non-PDPK, and tyrosine protein kinases (TPK) [123]. So far, many kinases have been reported to assist in tau phosphorylation. Microtubule-affinity regulating kinase (MARK) [124], p38 mitogen-activated protein kinases (MAPKs), Fyn, glycogen synthase kinase 3 (GSK3) [125], cyclin-dependent kinase 5 (CDK5) [126], c-Abl tyrosine kinase [127], c-Jun N-terminal kinase (JNK) and extracellular signal regulated kinase 2 (ERK2) [128] were all reported to have been involved in the hyperphosphorylation of tau (Fig. 4). Yet the numerous tau kinases participate in a rather complicated manner with no obvious key regulator and are usually shared by other non-tauopathy pathways.

Nevertheless, the complication of tau kinases activation network did not discourage AD researchers from seeking for potential small molecule drugs in tau kinase inhibitions. One of the earliest small molecule drug candidates that is believed to be involved in the tau kinase pathways and entered clinical trial for AD was the GSK-3β-inhibiting valproate, an approved drug that attempted a repurposing from epilepsy, bipolar disorder, and migraine treatment. In 2011, the clinical report published by Triot et al. announced that after dosing the patients with 10–12 mg/kg valproate for 24 months followed by a 2 month period of single-blind placebo treatment, valproate delivered no prevention in patient conditions of cognition, agitation or psychosis, and in contrast, is associate with acute toxicity [129]. Tideglusib, another GSK-3β inhibitor currently under evaluation, has been proved able to reduce tau phosphorylation as well as improve behavioural deficits in transgenic mice after three months of oral administration of 200 mg/kg [130]. In 2015, a 26 week, multi-centre, double-blind, randomized, placebo controlled, parallel-group, phase 2 study of tideglusib demonstrated to 306 mild to moderate AD patients with two oral doses (500 or 1000 mg/day) was evaluated and published [131]. The trial demonstrated that tideglusib is well-tolerated and without clinical efficacy. The other tau kinase small molecule inhibitors being evaluated clinically include the Abl-inhibiting nilotinib and Fyn-inhibiting saracatinib, in phase 3 trial and the re-evaluation for the Parkinson’s disease respectively (Table 3). Nevertheless, there is also a series of tau kinase inhibitors being evaluated preclinically as more tau-phosphorylating kinases are being identified. The thousand-and-one amino acid kinases (TAOKs) is a kinase family with three family members: TAOK1(PSK2), TAOK2(PSK1) and TAOK3(PSK3). Mass spectroscopy identified 44 and 41 human tau phosphorylation sites by TAOK1 and TAOK2 respectively, and demonstrates that TAOKs phosphorylation activities resulted in 28 hyperphosphorylated residues on tau that also correlates with the PHF-tau extracted from AD brain tissues [132]. In 2017, two small molecule inhibitor compound 43 (TAOK1 IC_50_ = 11 nM, TAOK IC_50_ = 15 nM) and compound 63 (TAOK1 IC_50_ = 19 nM, TAOK IC_50_ = 39 nM), that target both tau TAOKs have been reported [97]. Decreased tau phosphorylation in murine and human neurons of tauopathy by compound 43 inhibition indicates that the small molecule bears the potential to be further trialled in animal models [96]. However, so far there is no evidence to indicate that these small inhibitors may impact human brains in vivo.

**Table 3:** Evaluation of the tauopathy-related small-molecule drugs

Name	Molecular structure	Mechanism	Status
Tau aggregation inhibitors			
Oleocanthal		Bind to tau lysine residues	Preclinical
Dimethylfumarate		Bind to tau cysteine residues	Preclinical
Baicalein		Inhibit heparin-induced tau aggregation by initializing tau oligomer formation	Preclinical
Rosmarinic acid		Block the formation of the steric zipper structures essential for the β-sheets	See *Melissa officinalis* extract [94]
Methylene blue		Mediate tau cysteine oxidation	Succeeded by LMTX
LMTX			None of its three phase 3 trials and one 2/3 trial provided significant outcome
ACI-3024		Disaggregation of NFTs	Completed the first phase 1 trial in 2020 then remains inactive^a^
OLX-07010 [95]	Unpublished	Prevention of tau aggregation’s initiation	First phase 1 trial expected to reach primary completion in 2024
Tau kinase inhibitors			
Valproic acid		GSK-3β inhibition	Failed to meet endpoint in phase 3 trial completed in 2009
Tideglusib		GSK-3β inhibition	Failed to provide significant outcome in phase 2 trial completed in 2012
Nilotinib		Abl inhibition	Phase 3 trial expected to complete in 2026
Saracatinib		Fyn inhibition	Discontinued in 2018 after phase 2 trial due to lack of efficacy and re-entered trial for Parkinson’s Disease
Compound 43 [96, 97]		TAOK inhibition	Preclinical
Compound 63 [97]		TAOK inhibition	Preclinical
Tau kinase competitors			
Thiamet-G		OGA inhibition	Preclinical. Lack prolonged effect
MK-8719			Preclinical
LY3372689			In phase 2 trial
ASN90			Orphaned in 2018 after phase 1 trial then repurposed for diagnostic PET imaging
ASN51 [98]	Unpublished		Phase 2 scheduled in 2024
BIIB113 [99]	Unpublished		Phase 1 completed in 2023. Results not released
Microtubule stabilizers			
Epothilone D		Reduce tau toxicity by binding to tubulin and stabilize microtubule	Phase 1 trial completed in 2013 then discontinued^a^
TPI 287			Remains inactive after phase 1 trial for AD began in 2013 and was reported to induce strong allergic reactions in patients with primary four-repeat tauopathy

^a^There was no official conclusion announced behind the inactive or discontinued cases

#### Tau Kinase Competitors

An unconventional target in AD tauopathy leads the road back to the impaired brain glucose metabolism. O-linked N-acetylglucosamine (O-GlcNAc) transferase (OGT) and O-GlcNAcase (OGA) specifically mediate the reverse process on the same substrates: while OGT installs O-GlcNAc onto serine or threonine hydroxyl group, OGA removes it [133]. Together, the two enzymes make O-GlcNAc a reversible post-translational modification [134]. The OGT/OGA pair has reported a diverse range of more than 1000 reported substrates, and it has been confirmed that tau protein is also one of them [135].

O-GlcNAcylation affects AD brain activities both through the regulations of brain glucose uptake behind the blood–brain barrier [136] and through the competition with tauopathy [137]. Forebrain OGT loss in murine models led to decreased glycosylation and progressive neurodegeneration [138], which provides direct evidence of OGT/OGA’s pathological impact on cognition. It is hypothesised that the glycosylation and phosphorylation on tau are reciprocal upon shared residue sites, so that OGT’s activity may block the access of the tau kinases, reducing the chance of hyperphosphorylation during the process (Fig. 4) [139]. However, so far how the OGA/OGT-mediated glycosylation level is altered in AD brains remains controversial. In 1995, Griffith et al*.* described O-GlcNAc’s upregulation in AD brains compared with the control group of post-mortem human brain samples and points out such elevation is more significant in the front lobe, parietal lobe, and hippocampus [140]. In 2009, Liu et al*.* reported the remarkable downregulation of O-GlcNAc in AD cerebrum and demonstrated that the suppressed level is directly linked to AD tauopathy along with PP2A downregulation [137]. Again in 2015, Forster et al*.* detected O-GlcNAc’s upregulation in AD brains compared with their age-matched controls, which they also claimed to be the result of the unaffected OGT level and a 75% decrease in OGA protein level [141]. Despite the controversy surrounding its expression levels, OGA’s status as a potential tauopathy target became more convincing when studies revealed that the oral administration of an OGA inhibitor thiamet-G to Sprague Dawley rats effectively reduced their brain tau phosphorylation level in vivo [142]. Thiamet-G also does not alter normal tau phosphorylation after administration [143] however, possibly due to its activating effect on the tau phosphorylation kinase GSK-3β, the small inhibitor lacks prolonged effect [144]. To replace thiamet-G, its fluorinated derivative MK-8719 was later developed by medicinal chemistry and introduced as an OGA inhibitor by Selnick et al*.* in 2019 [145]. The compound still awaits more preclinical evaluations in vivo. The other OGA inhibitors developed for AD treatment include ASN51, ASN90, LY3372689 and BIIB113. Except for ASN90 which was orphaned after an unpublished phase 1 clinical trial in 2017 and announced re-evaluation for radiolabelling and diagnostic PET imaging purpose by its developer Asceneuron, the rest of the OGA inhibitors are still in clinical trials of different stages until more clinical results in AD patients become available to the public.

#### Microtubule Stabilizer

Another independent tau-related AD drug design concept is to directly rescue microtubule assembly from tauopathy-induced neurotoxicity by tubulin-binding and stabilisation. The strategy of microtubule-targeting agent was originally applied in chemotherapy, with two families of taxol-domain binders available: the taxanes and the epothilones. Both taxanes and epothilones dock to the identical binding site of the αβ-tubulin heterodimer subunit to decrease its disassociation rate. Epothilone D was originally discovered among a class of secondary metabolites in myxobacteria sorangium cellulosum and was later introduced as a microtubule-targeting brain-penetrant. The small drug is essentially uninvolved in the tau assembly process, however, was claimed to rescue neurotoxicity and restore cognition in tau transgenic mice [146]. The taxanes on the other hand, are known to display poor brain availability and requires the aid of drug delivery systems to cross the blood–brain barrier. TPI-287 as a third-generation taxane derivative however, successfully overcome this disadvantage and became another CNS-penetrating drug candidate [147]. The mechanism and brain availability of the microtubule stabilisers inevitably raised the question of drug-induced neurotoxicity, particularly since the epothilome or taxane-induced peripheral neuropathy is not uncommon among cancer patients [148]. Entering the phase 1 clinical trial in 2013, TPI-287 was quickly discontinued due to its severe side effects. The trial eventually reached official completion in 2019 and the drug has not re-entered any other dementia-related evaluations. The case of epothilone D on the other hand, was also inactivated after the completion of its phase 1 trial in 2013(result not published).

## Protein Phosphatases

### Dephosphorylation of AD Tau

Contrary to the complicated activation network of numerous tau kinases, the phosphatases responsible for the dephosphorylation of the altered taus in its dynamic counterbalance, on the other hand, can be simplified into a much shorter story. Despite the size of the Ser/Thr protein phosphatase superfamily, only three phosphoprotein phosphatase (PPP) class members PP1, PP2A [149] and PP2B [150] can dephosphorylate tau and are confirmed activated in AD brains. In vitro study has confirmed the abnormally phosphorylated sites including tau’s Ser 199, Ser 202, Ser 396 and Ser 404, excluding Ser 46 and Ser 235, were successfully dephosphorylated by PP1, PP2A and PP2B [151]. Among the three tau phosphatases, PP1 and PP2A together account for over 90% of dephosphorylation activity in various tissue types [149], putting PP2B in the position of a minor regulator. PP2A’s dominant role and brain-abundance is also to be noted. In rabbit tissue homogenate protein phosphatase assays, Cohen et al. reported the PP2A activity level to be 3.3–3.4 times higher compared with both brain PP1 and PP2B. Moreover, PP2A activity level in the brain tissue is measured to be 1.7 times higher than that of heart muscles, followed by a dramatic drop in skeletal muscles, liver and adipose tissues [152]. While PP2A alone accounts for a predominant portion of the total brain phosphatase activity [153], it also regulates PP1 activity by dephosphorylating the endogenous inhibitor I-1/DARPP-32 [154]. So far, PP2A is considered the key regulator of tau dephosphorylation in AD tauopathy and directly impacts the brain tau oligomerisation. In maintaining the counterbalance, PP2A’s activity level is comparable to the sum of the tau kinases. It has been reported that by selective inhibition of PP2A using the highly PP2A-specific marine toxin okadaic acid (OA) (selectivity PP2A: PP1 > 1000), hyperphosphorylation and significant accumulation of pathological tau was observed on harvested rat brain slices as a result [155]. However, unlike tau’s established brain-specificity, the PPPs are widely expressed across the human body and has been found playing an essential role in various non-CNV vital organs. For example, PP2A is known to be actively involved in the pathology of cardiac disease and injuries [156]. PP1 on the other hand, is heavily expressed in skeletal muscles due to its glycogen regulation functionality reciprocal to that of glycogen synthesis and breakdown [157].

### PP2A: An Underrated Target

PPPs have been long disregarded as targetable markers due to their vital roles and low tissue specificity. However, when disassembled and analysed separately, many subunit isoforms of PP2A are tissue-enhanced or even specific. PP2A has three subunits: the scaffolding subunit A, the regulatory subunit B and the catalytic subunit C. Subunit A and C of PP2A assembly both have two isomers α and β, with the α type being the more abundant form respectively. Among 55 tissue types and 6 blood cell types analysed and reported by Human Protein Atlas database, the normalised RNA expression level of PP2A Cα indicates its low tissue-specificity. The analysis of PP2A Cβ however, points out its highest RNA level in cerebral cortex followed by thalamus and white matter. While PP2A Aβ is highly liver specific, PP2A Aα is also found abundantly expressed in cerebral cortex, followed by a variety of brain tissues including hippocampal formation, thalamus etc. PP2A subunit B on the other hand, has been reported to exist in various isoforms that can be divided into four families: B (PR55), B′ (PR56/61), B″ (PR72/130) and B‴ (PR93/110). Subunit B regulates the substrate specificity and the activation of PP2A. As a result, several identified isoforms of PP2A subunit B are reported to be highly tissue specific. This includes the skeletal muscle specific B″α and B″β, pancreas enhanced BΔ, and brain enhanced Bβ, Bγ, and B′β. The cDNA microarray study of AD and non-AD gene expression levels of over 7000 genes highlighted that the significantly decreased level of PP2A catalytic subunit Cα in AD brain tissues compared with the non-AD controls [158]. Probed by mRNA level, a 63, 47, and 52% loss of PP2A Cα, PR61ε (B′ε) and the highly brain-enhanced PR55γ (Bγ) in AD hippocampus was also observed and reported [159], while the expression levels of PR55α (Bα) and PR61δ (B′δ) are of no significant change. Notably, no change was observed in the total PP2A or Bα subunit density in human cortex immunostaining result collected from human patient donors with Parkinson’s disease or Lewy body dementia [160].

The altered expression levels of PP2A subunits are directly associated with the PP2A activity level in the AD brains. Analysis of the PP2A dephosphorylation activity in brain homogenates observed a decreased activity level by 40% and 60% in frontal and temporal respectively, with no significant change in cerebellum compared with the control subjects [153]. While genetically engineered PP2A knockout cannot be achieved due to its fatality, reducing PP2A activity by selective inhibition, has led to the accumulation of pathological tau [154, 155]. The important role of PP2A in AD tauopathy and etiology has advertised its potential as a druggable target in AD. Its supressed expression level may provide an identifiably diminishing signal in AD diagnostic PET imaging for early-stage diagnosis. To develop PP2A-specific drug candidates, many studies have reported different PP2A small molecule inhibitors based on a library of various PP2A natural inhibitors discovered and reported as toxins and extracts [161–165]. For example, the well-studied and SAR-screened druglike cantharidin analogues (namely LB-100) [166] provides an excellent template for the development of radiolabelled PET tracers to probe the brain PP2A activity in vivo. However, so far, the application of these molecules only aligns with the need for potential anticancer agents and cardiovascular disease studies. Hence in this work, we would like to propose the PP2A-specific inhibitors’ potential as the future imaging probe in AD diagnostic PET. By administrating a radiolabelled PP2A-specific tracer, the diminished signal of PP2A in human brains in vivo may serve as an alternative approach to the diagnosis of early-stage AD type dementia.

Targeting PP2A as a therapeutic biomarker, however, must make a detour to the enhancement of PP2A activation and the prevention of PP2A endogenous inhibition. In 2010, Corcoran et al*.* reported sodium selenate as a PP2A exogenous agonist that selectively activates PP2A heterotrimers composed of the PR55 B subunit form of PP2A but not PP1. Compared with the vehicles, after the treatment with sodium selenate, the level of phosphorylated and total tau in the hippocampus and amygdala of the transgenic murine model overexpressing human tau441 have been lowered [167]. In 2016, results of a 24 week double-blinded randomized clinical trial dosing forty mild AD patients with up to 30 mg sodium selenate per day was published and the drug subsequently was announced as well-tolerated [168]. In 2022 and 2023 respectively, Vivash et al*.* published a phase 1b trial in behavioural variant frontotemporal dementia [169] and a phase 2 trial in chronic drug-resistant temporal lobe epilepsy [170]. However, no further information regarding its efficacy in AD patients is available. The therapeutic strategy of activating brain PP2A in AD patients via small molecule exogenous activators, so far, remains largely unexplored.

Nevertheless, the activity of PP2A is also affected by multiple endogenous regulators. The methylation of PP2A subunit C and B is co-regulated by leucine carboxyl methyltransferase 1 (LCMT1) and phosphatase methylesterase 1 (PME1). In 2016, Nicholls et al*.* reported that the overexpression of LCMT1 desensitised Aβ’s impact on cognitive impairment in mice, a phenomenon that could be attributed to rescued downstream tau function, and the sensitised outcome in the PME1 overexpression model [183]. In 2020, the opposite outcome was observed in murine models with reduced expression of the two enzymes [184]. PP2A in an inactive conformation stabilised by PME1 can subsequently be re-activated by serine/threonine-protein phosphatase 2A activator (PTPA). In contrast, CIP2A, ANP32a (I_1_PP2A) and SET (I_2_PP2A), ANP32b (I_3_PP2A) are identified as the cellular inhibitors of PP2A. Theoretically, the inhibition of CIP2A, ANP32a, ANP32b, SET, and PME1, or the enhancement of LCMT1 and PTPA, may all benefit brain PP2A level subsequently rescue the hyperphosphorylation of tau (Table 4). So far, not many studies have been conducted to explore the possibility of reducing tau hyperphosphorylation by assisting the activation of PP2A as a therapeutic approach. However, attempting PP2A reactivation via the inhibition of its endogenous inhibitors as an anti-cancer strategy is not an uncommon approach [185]. As the tau-targeting PET tracers being re-evaluated and approved, the PP2A activation approach may serve as the new direction of AD therapeutic research in the coming future.

**Table 4:** PP2A-modulating strategies with AD diagnostic and therapeutic potential

Target	Mechanism	Potential small molecule drugs
PP2A	PP2A agonistic binding to boost PP2A activity level as an AD therapeutic approach	Sodium selenite [167, 168]
PP2A subunit Aα	PP2A activation via binding of Aα subunit as an AD therapeutic approach	Phenothiazine [107, 171], tricyclic anti-cancer agents [172]
PP2A subunit C	PP2A-specific inhibitory binding as a method to probe AD pathological change and diagnostic PET	Cantharidin [166], okadaic acid [173] and microcystin [162] derivatives
PP2A subunit Bγ	Highly brain-enhanced PP2A subunit with suppressed expression level in AD tauopathy[159]. Potential target for AD pathological study and diagnostic PET	–
CIP2A	PP2A reactivation by targeting endogenous inhibitors as a therapeutic approach	Bortezomib [174], Celastrol [175], Erlotinib [176]
SET		Ceramide [177], COG112 [178], FTY720 [179]
ANP32a/b		Spermidine [180]
LCMT1	Potential therapeutic opportunity by LCMT1 enhancement	–
PME1	Potential therapeutic opportunity by PME1 suppression	ABL127 [181], AMZ-30 [182]
PTPA	Enhancing PTPA chaperon activity to elevate active-state PP2A as a therapeutic approach	–

## AD Immunotherapy

In recent years, AD immunotherapy, a therapeutic strategy that is independent from the conventional small-molecule drug discovery, is gaining more and more attention. The strategy applies synthetic peptide or monoclonal antibodies to trigger an immune response in the patients to help clear out the pathological target. By mechanism, AD drug candidates in this category can be divided into two groups: the passive immunotherapy antibodies and the active immunotherapy vaccines.

So far two AD immunotherapy drugs, aduhelm and leqembi, have received FDA approval and both fall into the passive immunotherapy category. Receiving the FDA approval and the EMA rejection in 2021, the fibrillar-amyloid-binding aduhelm became the first marketable AD immunotherapy drug and the first amyloidosis/tauopathy-targeting AD therapeutic as well. Leqembi on the other hand, was first developed by Englund et al*.* and reported in 2007 as a promising diagnostic detection tool for Aβ protofibril quantification without interfering the monomers or APPs [186], later was recognised as a highly selective novel Aβ protofibril reducer. The antibody received full FDA approval in 2023. The other two monoclonal antibodies in late stage of phase 3 clinical trial are donanemab and remternetug. Both drugs target AD-related amyloidosis with donanemab already applying for FDA approval in 2023. However, the controversy of amyloid-related imaging abnormalities (ARIA) follows these monoclonal antibodies ever since the early trials of bapineuzumab. The occurrence of ARIA-E in phase 3 trial participants is 25–36% (dose-dependent) by aduhelm [187], 12.6% by leqembi [188], and 24.0% (symptomatic and asymptomatic) by donanemab [189]. Other monoclonal antibody drug candidates currently in phase 1 or 2 clinical trials include ABBV-916, ACU193, MEDI1814, PRX012, trontinemab etc., targeting AD-related amyloidosis, and APNmAb005, bepranemab, E2814, JNJ-63733657, MK-2214, PRX005 etc*.*, targeting AD tauopathy (Table 5).

**Table 5:** Evaluation of the amyloidosis/tauopathy-related AD immunotherapies

Name	Mechanism	Target	Status
Aβ-targeting AD immunotherapies			
AAB-003	Passive, monoclonal antibody	Aβ	Discontinued after phase 1 trial in 2014. No change in cerebral spinal fluid biomarker levels were observed [191]
ABBV-916	Passive, monoclonal antibody	Aβ	In phase 2 trial. Estimated completion in 2031
ACU193	Passive, monoclonal antibody	Aβ	In phase 2/3 trial. Estimated completion in 2031
Aduhelm	Passive, monoclonal antibody	Aβ	Received FDA approval in 2021. Discontinued by Biogen in 2024^a^
Bapineuzumab	Passive, monoclonal antibody	Aβ	Discontinued during phase 3 trials in 2012 due to lack of benefit and observed ARIA. The first AD immunotherapy drug to enter clinical trial
Crenezumab	Passive, monoclonal antibody	Aβ	Discontinued in 2022 after phase 3 trial. Results available [192]^a^
DNL919	Passive, monoclonal antibody	Aβ	Discontinued in 2023 after phase 1 study^a^
Donanemab	Passive, monoclonal antibody	Aβ	Applied for FDA approval in 2023
Gantenerumab	Passive, monoclonal antibody	Aβ	Succeeded by trontinemab
GSK933776	Passive, monoclonal antibody	Aβ	Published phase 1 AD study in 2014 [193] then repurposed for age-related macular degeneration^a^
Leqembi	Passive, monoclonal antibody	Aβ	Received FDA approval in 2023
LY2599666	Passive, monoclonal antibody	Aβ	Discontinued in 2017 after phase 1 trial due to low target engagement
MEDI1814	Passive, monoclonal antibody	Aβ	Remains inactive since completion of phase 1 trial in 2016. Results available on clinicaltrials.gov^a^
Ponezumab	Passive, monoclonal antibody	Aβ	Discontinued in 2016 after failing phase 2 trial. Results available on clinicaltrials.gov
PRX012	Passive, monoclonal antibody	Aβ	In phase 1 trial
Remternetug	Passive, monoclonal antibody	Aβ	In phase 3 trial to be completed in 2025
SAR228810	Passive, monoclonal antibody	Aβ	Discontinued in 2018 after phase 1 trial^a^
Solanezumab	Passive, monoclonal antibody	Aβ	Discontinued in 2023 after phase 3 trial due to lack of cognitive preservation [194]
Trontinemab	Passive, monoclonal antibody	Aβ	New formulation of gantenerumab. In phase 1/2 trial till 2027
ABvac40	Active, vaccine	Aβ	Phase 2 trial completed in 2023. Results not published
ACC-001	Active, vaccine	Aβ	Discontinued in 2013 after phase 2 trial. Notably the trial was conducted with adjuvant QS-21 co-administration. Results available on clinicaltrials.gov^a^
ACI-24	Active, vaccine	Aβ	Currently in phase 2 trial for both AD and Down’s syndrome. Trial will last till 2026
Affitope AD02	Active, vaccine	Aβ	Discontinued after the 2013 phase 1 trial failed to meet endpoints
ALZ-101	Active, vaccine	Aβ	In phase 1 trial till 2025
AN-1792	Active, vaccine	Aβ	The first AD vaccine. Discontinued after phase 2 trial completed in 2003. Both antibody response and antibody binding are low [195]
AV-1959	Active, vaccine	Aβ	In phase 1 trial till 2026
CAD106	Active, vaccine	Aβ	Remains inactive since phase 2 trial in 2012^a^
Lu AF20513	Active, vaccine	Aβ	Phase 1 trial terminated in 2019. Results not available^a^
UB-311	Active, vaccine	Aβ	Remains inactive since completion of phase 2 trial in 2018. Results available[190]^a^
Tau-targeting AD immunotherapies			
APNmAb005	Passive, monoclonal antibody	Tau	Phase 1 trial expected to complete in 2024
Bepranemab	Passive, monoclonal antibody	Tau	In phase 2 trial till 2025
BIIB076	Passive, monoclonal antibody	Tau	Discontinued in 2022 after phase 1 trial^a^
E2814	Passive, monoclonal antibody	Tau	In phase 1/2 trial till 2027
Gosuranemab	Passive, monoclonal antibody	Tau	Discontinued after aborting phase 2 trial in 2021 due to lack of efficacy [196]
JNJ-63733657	Passive, monoclonal antibody	Tau	In phase 2 trial till 2025
Lu AF87908	Passive, monoclonal antibody	Tau	Phase 1 trial completed in 2023. No results published
MK-2214	Passive, monoclonal antibody	Tau	In phase 1 trial till 2025
PNT001	Passive, monoclonal antibody	Tau	Phase 1 trial completed in 2023 and results published [197]
PRX005	Passive, monoclonal antibody	Tau	Phase 2 trial expected to complete in 2027
RG7345	Passive, monoclonal antibody	Tau	Discontinued in 2015 after phase 1 trial^a^
Semorinemab	Passive, monoclonal antibody	Tau	Completed phase 2 trial in 2023. Published results demonstrated no change on tauopathy progression [198]
Tilavonemab	Passive, monoclonal antibody	Tau	Discontinued in 2021 after failing phase 2 trial
Zagotenemab	Passive, monoclonal antibody	Tau	Discontinued in 2021 after failing phase 2 trial
AADvac1	Active, vaccine	Tau	Phase 2 trial completed in 2019. Results published in 2021 [199]
ACI-35	Active, vaccine	Tau	In phase1/2 trial in 2023

^a^There was no official conclusion announced behind the inactive or discontinued cases

The active immunotherapies of AD, or the AD vaccines, are artificial antigens that stimulate human immune cells to clear the pathological targets. The amyloidosis-targeting AD vaccines currently being clinically evaluated include ABvac40, ACI-24, ALZ-101, AV-1959 and UB-311. Notably ARIA-E associated with the vaccines has not be observed in the reported trials, whilst asymptomatic ARIE-H is still reported [190]. The tauopathy-targeting AD vaccines being clinically evaluated are AADvac1 and ACI-35 (Table 5).

Notably, compared with the AD clinical trials before 2015 (i.e. small-molecule drug tideglusib; monoclonal antibody bapineuzumab; vaccine AN-1792), in these more recent trials, the focus of trial criteria is more inclined to include AD-MCI patients to evaluate the preventive effects (i.e. small-molecule drug efavirenz; monoclonal antibody crenezumab, leqembi; vaccine ABvac40), which may also affect the efficacy, comparability and significance of the reported clinical data. Apart from the amyloidosis/tauopathy-targeting mechanism, there are also a number inflammation and combined mechanism antibodies (i.e. AL002, pepinemab, gamunex, IBC-Ab002) and vaccines (i.e. CpG ODN, protollin) being developed and assessed. Since this paper mainly aims to discuss the development, evaluation and status of the conventional small-molecule drugs, these immunotherapy drugs will not be discussed in detail. So far, no tauopathy-targeting AD immunotherapy has reached the late stage of phase 3 clinical evaluation. The clinical performance of these AD immunotherapy drug candidates remains to be seen in the upcoming future.

## Conclusion

The PET tracers in AD diagnostic imaging started from the radiolabelled glucose analogue [^18^F] FDG based on dementia-impaired brain glucose metabolism, then progressed into the target-specific small molecules binding to Aβ and tau species with high selectivity over the two past decades, revealing more and more facts behind the AD neurotoxicity in vivo. Based on the amyloid cascade hypothesis, Aβ and tau targeting small molecule drugs with various inhibitory or modulatory mechanisms were developed and trialled yet so far has not achieved any breakthrough in AD cognitive improvement. The numerous failed small molecule drugs subsequently point out the limit of the amyloid cascade hypothesis and push forward the demand for AD-related target-specific imaging evaluations. Currently, the design of AD small molecule drugs is highly focused on reducing tauopathy-induced neurotoxicity with more and more tau kinases being introduced as new therapeutic targets. In this work we also discussed the essential role of PP2A as the key dephosphorylator of tau and proposed PP2A as a potential new biomarker in AD diagnostic imaging and small molecule drug discovery in the future.
